# Supplementary material for: LPS-Induced Garcia Effect and Its Pharmacological Regulation Mediated by Acetylsalicylic Acid: Behavioral and Transcriptional Evidence
Source: Biology (Basel). 2023 Aug 7;12(8):1100. doi: 10.3390/biology12081100 (PMC10451780; doi:10.3390/biology12081100)
Supplement: Supplementary file 1 [file biology-12-01100-s001.zip › biology-2465130-supplementary.pdf]

Supplementary Material

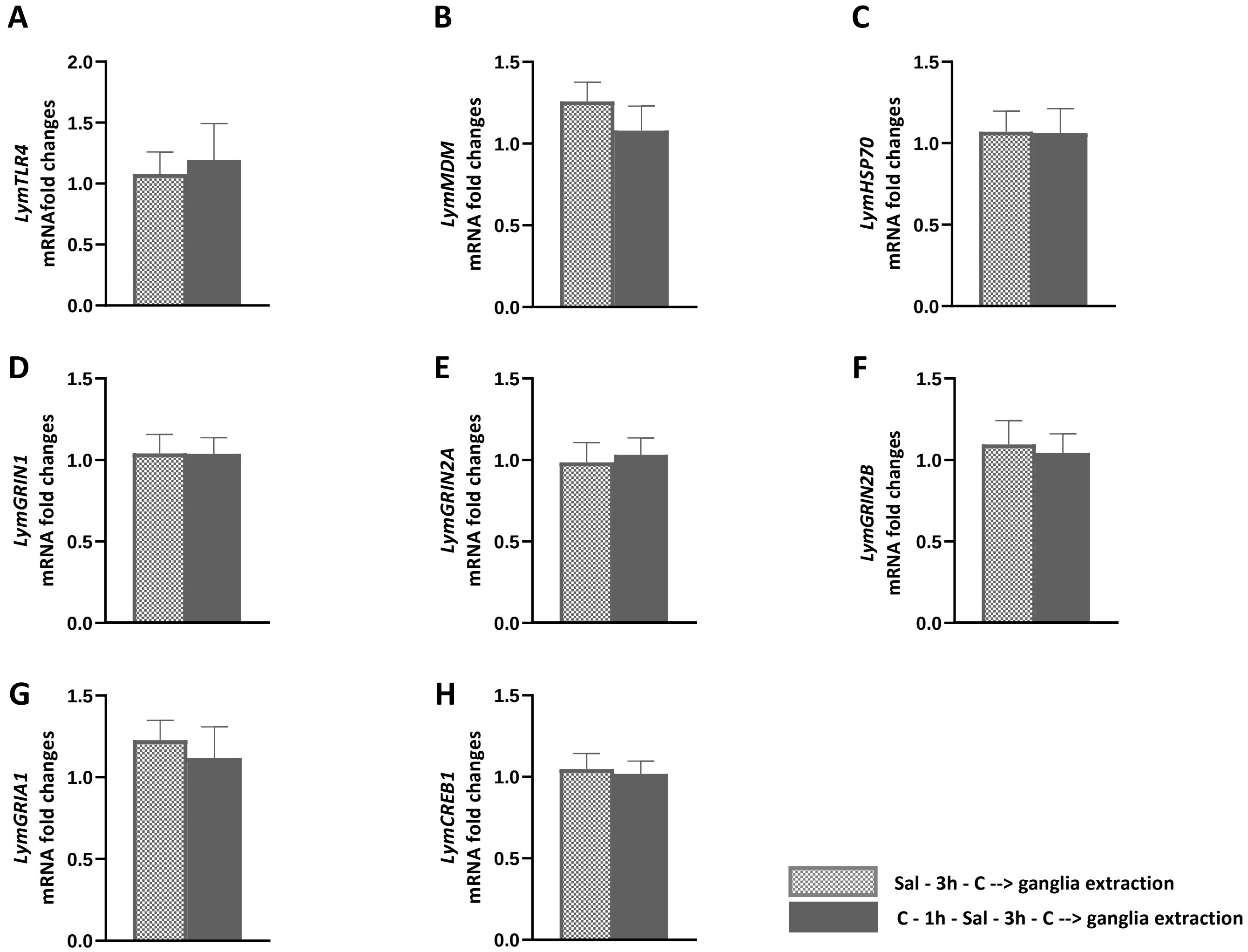

Figure S1. *LymTLR4*, *LymMDM*, *LymHSP70*, *LymGRIN1*, *LymGRIN2A*, *LymGRIN2B*, *LymGRIA1*, and *LymCREB1* expression levels showed no significant difference in snails exposed to carrot slurry 1 h before and 3 h after the snail saline injection (Saline group) and those of them exposed to carrot slurry only after the snail saline injection (Saline_C group). The expression of *LymTLR4* (**A**), *LymMDM* (**B**), *LymHSP70* (**C**), *LymGRIN1* (**D**), *LymGRIN2A* (**E**), *LymGRIN2B* (**F**), *LymGRIA1* (**G**), and *LymCREB1* (**H**) were measured in the central ring ganglia of snails injected with snail saline and then exposed for the first time to carrot slurry 3 h later (checkered bars) and those exposed to carrot slurry 1 h before and 3 h after being injected with snail saline (full dark grey bars). Immediately after the re-exposure to the carrot slurry for 2 min, snails of the Saline and Saline_C groups were sacrificed, the central ring ganglia were dissected, and the RNA was extracted and reverse-transcribed. No significant differences were found in the expression levels of these targets between the groups. The mRNA levels were analyzed by RT-qPCR. N = 8 for each group. Data are represented as means ± SEM and were analyzed with unpaired *t*-test.

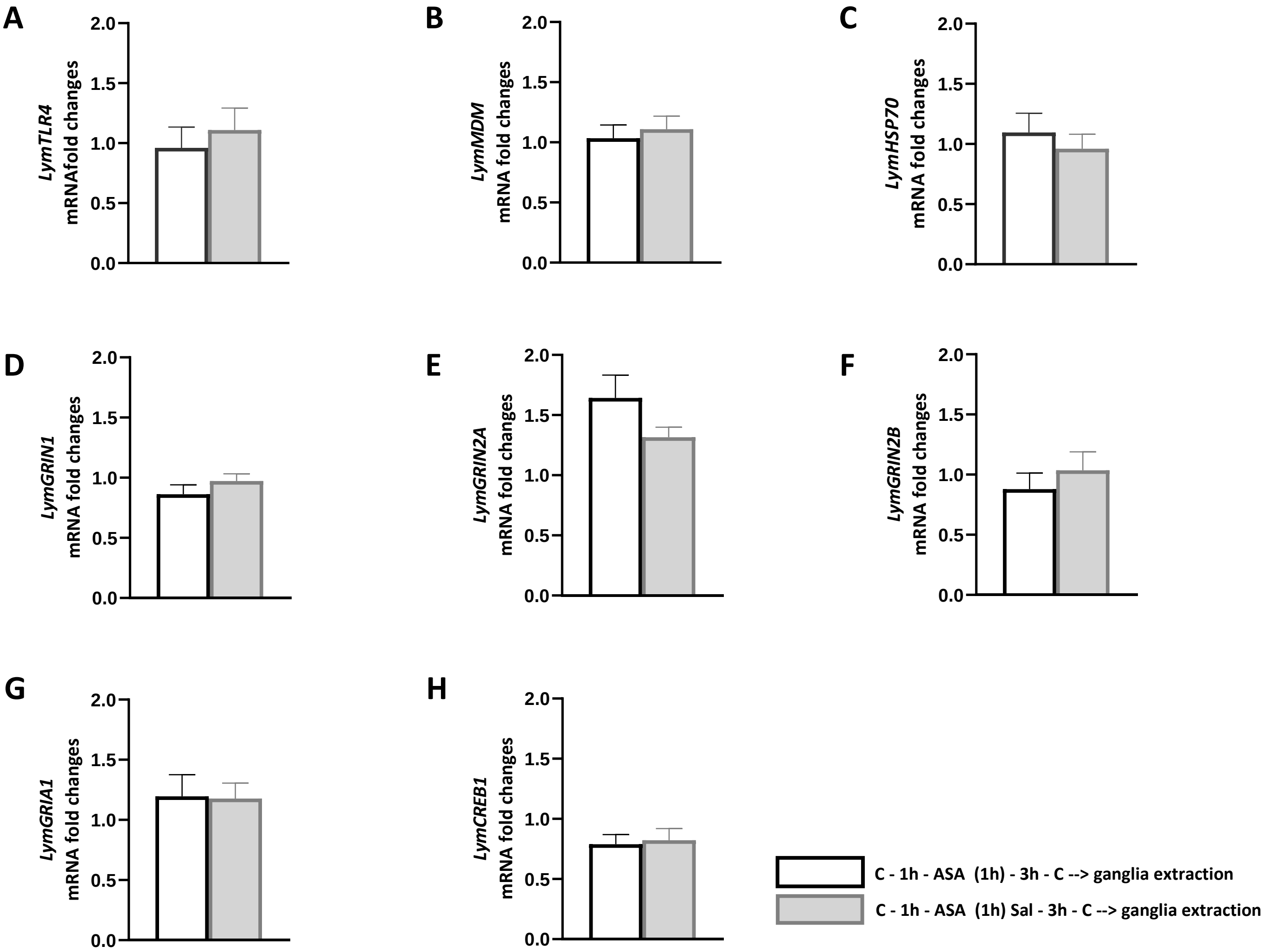

Figure S2. *LymTLR4*, *LymMDM*, *LymHSP70*, *LymGRIN1*, *LymGRIN2A*, *LymGRIN2B*, *LymGRIA1*, and *LymCREB1* expression levels showed no significant difference in snails of the ASA group and those of the ASA_Saline group. Snails of the 'ASA group' (white bars) were exposed to carrot slurry for 2 min and the number of rasps elicited by the carrot slurry was counted. One hour later, snails were exposed to ASA for 1 h. Three hours later, snails were re-exposed to carrot slurry for 2 min and the number of rasps elicited by the carrot slurry was again recorded. Snails of the 'ASA_Saline group' (full grey bars) were exposed to carrot slurry for 2 min and the number of rasps elicited by the carrot slurry was counted. One hour later, snails were exposed to ASA for 1 h and immediately after were injected with snail saline. Three hours later, snails were re-exposed to carrot slurry for 2 min and the number of rasps elicited by the carrot slurry was again recorded. Immediately after the re-exposure to the carrot slurry for 2 min, snails of the ASA and ASA_Saline groups were sacrificed, the central ring ganglia were dissected, and the RNA was extracted and reverse-transcribed. The expression of *LymTLR4* (**A**), *LymMDM* (**B**), *LymHSP70* (**C**), *LymGRIN1* (**D**), *LymGRIN2A* (**E**), *LymGRIN2B* (**F**), *LymGRIA1* (**G**), and *LymCREB1* (**H**) were measured and compared. No significant differences were found in the expression levels of these targets between the groups. The mRNA levels were analyzed by RT-qPCR. N = 8 for each group. Data are represented as means ± SEM and were analyzed with unpaired *t*-test.

To identify and characterize the transcripts codifying for the glutamate ionotropic receptor NMDA type subunits 2A and 2B

(GRIN2A and GRIN2B) and the glutamate ionotropic receptor AMPA type subunit 1 (GRIA1) in *L. stagnalis*, we recurred to the contig annotation table generated by Benatti et al., (2022) [60] (Table S1).

Table S1. Contig annotation table. For each contig, the ID, the contig length (bp), and the RefSeq protein ID identified in *Biomphalaris glabrata* (a gastropod whose genome and transcriptome have been characterized and annotated) are reported with the corresponding gene definition. When available, the RefSeq protein ID identified in *Mus musculus* and *Homo sapiens* is also reported, together with the gene symbol.

L. stagnalis **contigs**			**Homology association by BlastX**					
Contig ID	*Contig length (bp)*	*RNAseq TPM value in CNS*	*RefSeq protein ID B. glabrata*	*Gene symbol B. glabrata*	*RefSeq protein ID M. musculus*	*Gene symbol M. musculus*	*RefSeq protein ID H. sapiens*	*Gene symbol H. sapiens*
FCFB01224380.1	2477	3.080	XP_013076880	LOC106063108	NP_032196, XP_006521857	*Grin2a*	NP_001127879, NP_001127880, NP_000824, XP_016878662, XP_016878661, XP_011520763, XP_011520760	*GRIN2A*
FCFB01152357.1	2145	3.048	XP_013088398	LOC106072555	XP_006505638, XP_006505637, XP_011239523, XP_011239522, XP_006505636, NP_032197, XP_017176885	*Grin2b*	XP_011518930, XP_011518931, XP_016874708, NP_000825	*GRIN2B*
FCFB01164694.1	1345	10.009	XP_013069087	LOC106056784	NP_001034284	*Gria2*	XP_011535937, NP_001107655, NP_001244952, NP_001244951, XP_016864882, NP_001244949	*GRIA1*

This approach allowed us to identify one specific transcript per each glutamatergic receptor type: FX_180835 (*LymGRIN2A*), FX_180839 (LmGRIN2B), and FX_183516.1 (*LymGRIA1*). All transcripts resulted to be expressed (TPM > 0) in the publicly available *L. stagnalis* transcriptome (i.e., Transcriptome Shothgun Assembly).

To confirm whether these transcripts were expressed in the central ring ganglia of *L. stagnalis*, we performed qualitative PCR on each transcript using the set of primers listed in Table S2.

Table S2. Validated primers for sequencing. For each predicted target are reported the relative transcript FX_, the forward (FW), and reverse (RV) primers' sequences, with the corresponding position on the transcript.

**Match on**	**Predicted target**	**Position on transcript**		**Primer sequence**
FX180835	Putative *LymGRIN2A*	FW	2119	TAAGTTTAACTGGACGGACT
		RV	3645	ATAGCGTCCTTGACATTAGT
FX180839	Putative *LymGRIN2B*	FW	2439	CAGTATTGAGAACATGGTTG
		RV	4273	GGTTCCTTGATGGTTTATTA
FX183516.1	Putative *LymGRIA1*	FW	250	GGATCTTCTGACATCAAGTT
		RV	2477	CTGCAGAGAATGTACCAATA

We performed qualitative PCR using Dream Taq DNA polymerase (Thermo Scientific, Waltham, Massachusetts, United States) as previously described [60]. Primer sequences were designed by NCBI PrimerBLAST software (https://www.ncbi.nlm.nih.gov/tools/primer-blast/ (accessed on May, 31 st, 2023) and were synthesized by Merck KGaA (Darmstadt, Germany). PCR products were electrophoresed on agarose gel (2%), and DNA fragments were visualized by UV illumination to confirm the correct amplicon size. PCR products (600–800 bp) were purified using the High Pure PCR Product Purification Kit (Roche Diagnostics Corporation, Indianapolis, Indiana, USA) following the manufacturer's instructions, and were directly sequenced using the Sanger sequencing method. Sequencing was performed using ABI

PRISM 3130xl Genetic Analyzer (Thermo Fisher Scientific, Waltham, Massachusetts, USA) and BigDye Terminator v1.1 Cycle Sequencing Kit (Thermo Fisher Scientific, Waltham, Massachusetts, USA). The sequence analysis of the PCR fragments was performed using Sequence Scanner Software 2.0 (Thermo Fisher Scientific, Waltham, Massachusetts, USA), and sequences were compared and aligned to the contigs of *L. stagnalis* using BLASTn (https://blast.ncbi.nlm.nih.gov/Blastn/ (accessed on June 1st, 2023). The sequences generated by Sanger sequencing confirmed that the transcripts predicted in silico by the computational analysis uniquely matched their respective templates.

Moreover, we found that all transcripts contained an open reading frame (ORF) (Table S3) whose identity was further confirmed by aligning its sequence with the amino acid sequences of the corresponding enzyme from other invertebrate and vertebrate model organisms, like *B. glabrata, Aplysia californica, M. musculus,* and *H. sapiens* (Table S4). The amino acid sequences were predicted using the Open Reading Frame (ORF) Finder tool of NCBI (https://www.ncbi.nlm.nih.gov/orffinder/ (accessed on June 1st, 2023).

Table S3. ORFs of the putative glutamatergic receptors in *L. stagnalis*. For each contig, the ID, the FX_ value corresponding to *L. stagnalis* Transcriptome Shotgun Assembly, the ORFs, and the predicted amino acid size (aa) are reported.

		Lymnaea stagnalis	
TARGET	MATCH ON	**ORF**	**aa size**
LymGRIN2 A	FX180835	MATSMEENRVLILGGTGFIGRNLVDYLVRNKLARKIRVVDKVPPQMAWLNPRHLESFENPAVEFCQ<br>ANIINKASAEKVFCDDDGGFDFVINLAAETKHGQ<br>SEPVYKEGIYRLSLNCAELAAMHKVKRFIELSTAQIYTHEKKPAREDAKSEPWTNLARHKLDVEKAL<br>ENYVDLNYVILRPAIVYGIGDRNGLTPRLIIGA<br>IYKYTMQKMQMLWTKDLKMNTVHVDDVVRAIWHLCFHGNRGQVYNLTDKGDTTQGLISSLVSEL<br>FDINYDFVGAIFSNLARVRMTDIVDDINEKHMRPWS<br>DACQRDQVSNTPLNPFIDQELLYNKHLFIDGSKIESTGFAYTIPKLEIGHLKEILDDYITLGLMPRSLVS<br>NEVFYTPTDVEAACLDHGADGDCGLEGIDH<br>TADQDSAHS	409
LymGRIN2 B	FX180839	MEMISNPMDIPHSHQPATCLKIKLLWVDSNNLLQMSSQRFHCDALHGNFPVTPTTISRVVEPSIDATI<br>GRTCQCLKHAGAKPVLTSFTNIYSRAEKYSVQ<br>DQQHDAKEKSPRNKISTTGSMEFLASCPEKRWNSFLSCRGMCTHIGNLNAYFSFRFLSPERGALINVE<br>RLPQERVCKVEKMNSASCRGSNIFYKTKLNRQ<br>KSSYSVTSNIHFIPKEITGNISHSEYSTERKSSPGIRTCVDCKASFMKTSDTPCVGRFRFESSSDKSPKSLRS<br>HSDSHANIWSLDTNHCSSTPSTDTHAT<br>SSNTTASFPTKPTRTFEQRWRPTKIVSDYPSRVSTLLSFVLVFLTLVASQTNGQNIKAGIHAIYPDRISMS<br>KALSTELISTLYSVRRNLHVLRSVYDIE<br>HVNTSLKNDDPKEILNLFCKGVFPHHVTTILNINNPLGMKRRTSSNQYILELASYLGLPIISWDTEYIV<br>GSQSLRTVQLAPTIEHQAKAMVSMLERYNWT<br>AFTIVTGPVSGSSQFVTSIENMVEQSQKNALKTPAHSRKESLNILSIINIRKPEDILPQLVKVKGSDTRVF<br>LLHSASSMALDIIQTARTLNLTDKDYVWI<br>LTRTAIPNAREGPRSFPVGLMGIDFDFELTAMRQALKWGIMIWLNALADMAKTQGLLANMTIPPKF<br>SCDSDKEPFWRDGEILYRHMLKVNILGEPQIKFN<br>ENGTLQQTDLIIVNLQWIVGGKNKTEWKQVGRWTLHGLKMNEIVWPGESTVPPTGKPKRAFLRVA<br>TLNELPYVIYRNLSENGGCEEKSLPCQIYNRNDKK<br>EITSNVTIAKCCAGLSMDLLKILSDQLNFDYEIKEVIDGKWGVLINKTTNAWNGLVKALLNNEADIV<br>MTAFKINPERASAVNFSVPYLETGIKIIVALRD<br>GAISPTAFLEPYDYASWSLILIFSVHATGSSILIFEWLSPYGLNRGHTPMRDHKFSLFRSFWLIWAMLFS<br>TSVQTDTPKGIASRFLANIWALFALVFLAS<br>YTANLAAFMITKEEFYDLSGIQDYRLQNPYTMKPPFRYATIPNGSTEANIRANHKDMYNYMKAFNQ<br>PDVDAGIAELKQGRIQAFIYDSSVLEYYASRDPK	1504

		CRLVTVGNRYAMTGYGVGFPPNNKPSRNPWIDKFNKHILKLQENGDMDRLQKFWLAGACDTKEK GVSNRTLGILNFTSAFILLGSGVLLGLLILIFEHLYFKFCRKRLRKWDKCGCCALVSLSMGKSLQFKDY VDEAMSAYSKTRCKNP VCEIQIWKLRHQLDMALLKIDHLQNQLCEISGEFLPLMPSSTVENEKRTEHQQQKPLAVRANGMQQ SQHYKTEKSNHRQGKKTASKLPSQSAIDLPEVSF LRSLELRDGGNFIDKFEEYIENYEKPDSSPVADHFSDPLLPPPPQGESPLLGSSACNDDTGGGGSSYGG DTFLGFDDFHSENAGSRSASVLAGRMAAMGS SPGNVLRRTPSYTSAVGRDNGLDSASDSSPNMTGRVRQYDGKTYVGVDSSSTVT	
LymGRIA1	FX183516 .1	MMAKSHAKRIVVHSLIVMDQRFMIYCVIFALIGLHSTRSLERSVGLILQEDMPTKDTEYEQAFDFATM VVNSNLLNERRPGREMARVFPTLRNRTVFSDN YKLGGTICGMANEGVMVIVGTSRASSFNTIQSYCQALQVPYILVTPSRPNPSDGYHYDLSVCPPYIEAV MKVVANLSLADNKVFYVYDSDDGLWRLQRMY QYFQMKDIMPRVLDAFRVRDISKAYVILRALDKLSEEKTIVLDLSTTQAYKIILEQIVDVGMNRENYH YILAGADAMDLNLDRDFFSQFLYGGVEITAFQ FVNNQTDTYRKWQQMWEQYKDDFPNLFPLKTGSALMIDAVRALHEALMFEIPKQHTARTHPSRTC DMDNFKSSDVTSPLINSLNKIYFQGLTGPLALKQG RRSEYSIDVYKLGFKQPMAKQATWKSSDLMDGSFKTFVPEVAIQNTTQRVTTLIEPPFVMRIENRNGA PPVGPNLDLEGYCIDLIEALARSEDFEYQIYL TEEYGDKNETDGTWNGIIGQLINQERDIAVAPLTITQDRERVVDFSKPFMDTGISIMIKKPDKTKPGVF SFMDPLDTRVWLCIAIGFLAVSGVLYFVGRF SPYEWNVSEDSTERTATTVFSISNTLWFSLGALMQQGSDISPRSFSGRVIGSAWWFFTLIIISSYTANLA AFLTIEKLVVSISSADDLVGHPTIKYGTKK TGSSWRFFEKSTVDTFVRMRKEMLENADEVLFEDYSEGVRKVRESKGTYAFLLESAMNSYYSQQEPC DTMMVGDKLDNKGYGVATYVNYPLRHNINIAVL TLKEKGELIKLTQKWWFDKGQCGDQSVSKESTGTQSALTLSNVSGIFHILIGGLVLSMLTSSLEYLIQR KLRIRRKNIAKKATKNYTLPVPPPPPRRLCF GSAANRSNGIEPVWENGMSSDAAAAAAHEQQNLMSEDPDEYHNGT	945

As shown in Table S4, the predicted protein had several amino acids very close to those of the human protein and the corresponding orthologue of several organisms extensively employed as standard models in preclinical studies.

Table S4. ORF homology table. Homology between the ORF of the putative glutamatergic receptors in *L. stagnalis* with orthologues from different organisms. For each contig, the ID, the amino acid (aa) size (bp), the FX_ value corresponding to *L. stagnalis* Transcriptome Shotgun Assembly, and the RefSeq protein ID identified in *B.glabrata, A. californica, M. musculus,* and *H. sapiens,* along with the corresponding gene definition and aa size, are reported.

Lymnaea stagnalis			***Biomphalaria glabrata***			***Aplysia californica***			***Mus musculus***			***Homo sapiens***		
TARGET	MATCH ON	aa size	Accession	Target	% identities	Accession	Target	% identities	Accession	Target	% identities	Accession	Target	% identities
LymGRIN2A	FX180835	409	XP_013076878.1	*Predicted Glutamate receptor ionotropic, NMDA 2A-like*	51.12%	XP_012938956.1	*NR2A*	43.65%	P35436.2	*N-methyl-D-aspartate receptor 2A subunit*	39.71%	Q12879.1	*N-methyl-D-aspartate receptor 2A subunit*	32.48%
LymGRIN2B	FX180839	1504	XP_055875497.1	*Predicted Glutamate receptor ionotropic, NMDA 2B-like*	68.75%	NP_001191485.1	*NR2B*	58.99%	NP_001350679.1	*N-methyl-D-aspartate receptor 2B subunit*	33.92%	NP_000825.2	*N-methyl-D-aspartate receptor 2B subunit*	33.98%
LymGRIA1	FX183516.1	945	XP_013069087.1	*Predicted glutamate receptor-like*	83.00%	NP_001191398.1	*Glutamate receptor subunit protein GluR3*	78.00%	P23819.3	*AMPA-selective glutamate receptor 1*	39.00%	P42263.2	*AMPA-selective glutamate receptor 3*	36.97%

Candidate primers for quantitative RT-qPCR were designed with NCBI Primer-BLAST software (https://www.ncbi.nlm.nih.gov/tools/primer-blast/, accessed on June 1st, 2023) and synthesized by Merck KGaA (Darmstadt, Germany). Primers were designed to have a length of 19–23 nucleotides, a melting temperature between 58 and 60 °C, a GC content between 40 and 60%, and to generate an amplicon between 150 and 250 bp. All primers produced consistent results without amplifying off-target products or generating primer dimers (data available from the corresponding author upon request). Following amplification, each primer pair produced amplicons that yielded single bands at the correct size after electrophoresis in 2% agarose gels. No amplification was observed in cDNA from another pulmonated gastropod, *Pomacea canaliculate,* or controls that lacked reverse transcriptase in the RT-PCR (data available from the corresponding author upon request). Primer specificity was also checked by melt curve analysis (Figure S3). A single sharp peak with no primer-dimer was observed for all used primer pairs.

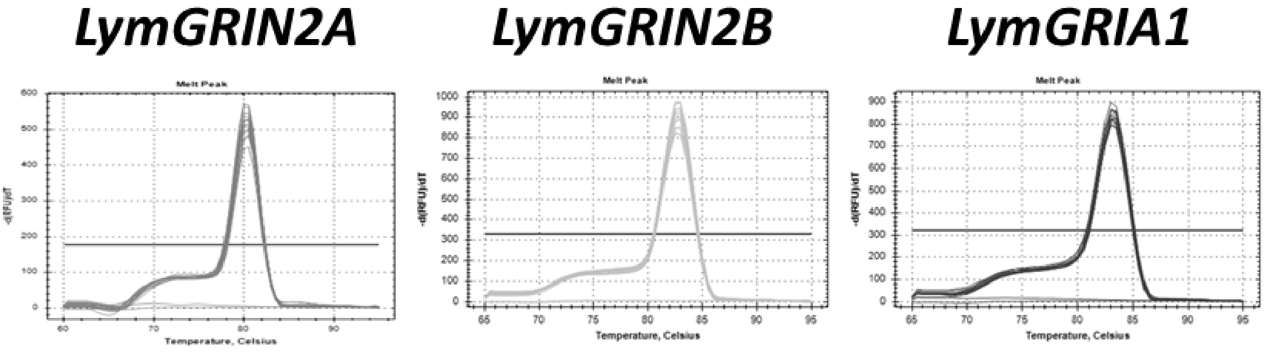

Figure S3. Melting curves analysis. For each target, a single sharp peak with no primer-dimer was observed.

Results showed that the RT-qPCR efficiency was between 97.98% and 105%, and the R2 of primers was greater than 0.98. In addition, the mean Cq of *LymGRIN2A*, *LymGRIN2B*, and LymGRIA ranged from 19.16 to 25.23 for 20 ng of cDNA with moderately abundant mRNA levels in the ganglia of *L. stagnalis* (Table S5).

Table S5. Validated primers for gene expression analysis. For each enzyme of the KP, the relative transcript FX_, the forward (FW) and reverse (RV) primers' sequences, with the corresponding size (bp), efficiency, R2 score resulting from the validation experiments, and the Ct value obtained with 20 ng of cDNA are reported.

Transcript	Target	Size (bp)	Primer sequences	Efficiency	R2	Ct value 20 ng
FX180835	*Lymnaea stagnalis NMDA-type glutamate receptor subunit 2A*<br>*LymGRIN2A*	129 bp (3454-3583)	FW: GATCACCAAGGATGATTACT<br>RV: CTTGGCTATATTCAAGTCTGT	105%	0.99	25.23
FX180839	*Lymnaea stagnalis NMDA-type glutamate receptor subunit 2B*<br>*LymGRIN2B*	126 bp (4147-4273)	FW: GACTCCTCTGTTTTGGAATA	97.98%	0.98	19.16

			RV: GGTTCCTTGATGGTTTATTA			
FX183516.1	*Lymnaea stagnalis AMPA-type glutamate receptor subunit 1* *LymGRIA1*	111 bp (1205-1316)	FW: AGACTGTTGTAGCTGTCCTT	99.85%	0.99	20.32
			RV: ATAGCTATTGGATTTCTTGC			
